# Diversity, Complexity, and Specificity of Bacterial Lipopolysaccharide (LPS) Structures Impacting Their Detection and Quantification †

**DOI:** 10.3390/ijms25073927

**Published:** 2024-03-31

**Authors:** Flavien Dardelle, Capucine Phelip, Maryam Darabi, Tatiana Kondakova, Xavier Warnet, Edyta Combret, Eugenie Juranville, Alexey Novikov, Jerome Kerzerho, Martine Caroff

**Affiliations:** 1 LPS-BioSciences, Bâtiment 440, Université de Paris-Saclay, 91400 Orsay, France; flavien.dardelle@lpsbiosciences.com (F.D.); maryam@lpsbiosciences.com (M.D.); eugenie.juranville@lpsbiosciences.com (E.J.); 2 HEPHAISTOS-Pharma, Bâtiment 440, Université de Paris-Saclay, 91400 Orsay, France; cp@hephaistos-pharma.com (C.P.); an@hephaistos-pharma.com (A.N.); jk@hephaistos-pharma.com (J.K.)

**Keywords:** endotoxin, lipopolysaccharides, LAL, TLR-4 activation test, 3-OH-fatty acids, endotoxin quantification

## Abstract

Endotoxins are toxic lipopolysaccharides (LPSs), extending from the outer membrane of Gram-negative bacteria and notorious for their toxicity and deleterious effects. The comparison of different LPSs, isolated from various Gram-negative bacteria, shows a global similar architecture corresponding to a glycolipid lipid A moiety, a core oligosaccharide, and outermost long O-chain polysaccharides with molecular weights from 2 to 20 kDa. LPSs display high diversity and specificity among genera and species, and each bacterium contains a unique set of LPS structures, constituting its protective external barrier. Some LPSs are not toxic due to their particular structures. Different, well-characterized, and highly purified LPSs were used in this work to determine endotoxin detection rules and identify their impact on the host. Endotoxin detection is a major task to ensure the safety of human health, especially in the pharma and food sectors. Here, we describe the impact of different LPS structures obtained under different bacterial growth conditions on selective LPS detection methods such as LAL, HEK-blue TLR-4, LC-MS^2^, and MALDI-MS. In these various assays, LPSs were shown to respond differently, mainly attributable to their lipid A structures, their fatty acid numbers and chain lengths, the presence of phosphate groups, and their possible substitutions.

## 1. Introduction

Lipopolysaccharides (LPSs) are amphiphilic glycolipids and the main components of the Gram-negative bacterial outer membrane [1]. Although they share a common architecture, their structures are complex and all different. This amazing diversity is due to the diversity of their major polysaccharide contents. They are called LPSs because they are made of a lipid moiety named lipid A [2], linked to an oligosaccharide core of about ten sugars, substituted by a long-chain polysaccharide. This polysaccharide extending outside the bacterium, called O-antigen, is made of repeating units of one to eight monosaccharides [3,4].

**Figure 1:**
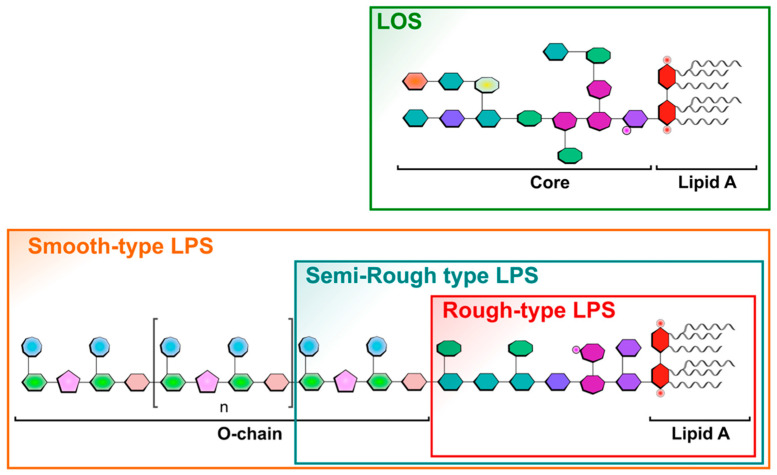
Schematic representation of different LPS forms and structures illustrating the different existing LPS types found in different bacteria, as in wild-type bacteria with Smooth-type LPSs versus LPSs found in bacterial mutant strains like the Semi-Rough-type or Rough-type LPSs. Lipo-oligosaccharide LOSs are found in specific bacteria as mentioned in the text. Sugars are symbolized by penta-, hexa-, or octagonal forms. Phosphate groups are illustrated by small red cycles, and FA by wave lines.

LPS O-chains are responsible for bacterial antigenicity and represent their serotypes. LPS biosynthesis has been well documented by R.C.H. Raetz group [5]. Molecular basis of LPS heterogeneity has also been described [6,7,8,9]. In some strains, because of the loss of the Wzy polymerase enzyme [10,11], a single O-unit is added to the core, and such LPSs are known as Semi-Rough-type LPSs (Figure 1). When the O-chain is absent due to the mutation of the WaaL ligase [12], the corresponding LPSs are referred to as Rough-type LPSs. Some LPSs naturally lack O-chains such as the highly virulent pathogens *Neisseria meningitidis* [13], *Neisseria gonorrhoeae* [14], *Bordetella pertussis* [15], *Haemophilus influenzae* [16], and *Campylobacter jejuni* [17]. In these species, as shown in Figure 1, the core moieties become responsible for antigenicity, and such LPSs are called LOSs for LipoOligoSaccharides [18].

LPSs are responsible for the impermeability of the bacterial outer membrane, thereby contributing to antibiotic resistance. They are the target structures of bacteriophages, components of the host’s immune system, and antimicrobial peptides [19,20,21].

LPSs can be released from bacteria either following death or during their multiplication, or in the formation of outer membrane vesicles (OMVs) [22,23]. Most released LPSs are highly potent molecules capable of eliciting beneficial or dose-dependent deleterious effects, in humans and animals. Humans are highly sensitive to those LPSs that behave as endotoxins, much more than rats, mice, or non-human primates but as sensitive as rabbits. At low doses, LPSs have a beneficial activity since they enhance the immune system responsiveness of the host. At higher concentrations, they stimulate an over-production of proinflammatory cytokines such as tumor-necrosis factor alpha (TNF-α) [24] or interleukins such as IL-1 and IL-6 [25] and can lead to sepsis or multi-organ failure [26]. This is why deleterious LPSs are called endotoxins. In short, all endotoxins are LPSs, but not all LPSs are toxic, i.e., not endotoxins. This is a significant difference and can lead to considerable confusion, since the terms endotoxin and LPS are often but erroneously used interchangeably [27].

LPS structural details exert a strong influence on the biological activities they induce. The number of fatty acids (FAs) and the length of their carbon chains, as well as the presence of phosphate groups and their possible substitution with various decorations [28], are crucial for the regulation of endotoxic activities. The lipid A structure is recognized in humans by the innate immune system through the LPS-LBP-CD14 cascade leading to the TLR4/MD2/LPS activating complex [29,30] in myeloid cells, notably macrophages and dendritic cells. This cascade results in the secretion of pro- and anti-inflammatory cytokines, thereby initiating inflammatory and immune-defense responses [31].

Substitution of the phosphate groups impacts LPS biological activities [32,33,34] as well as LPS binding to the positively charged polypeptide antibiotic, Polymyxin B [35,36]. It is important to remember this, considering that Polymyxin B columns are often used to detoxify pharmaceutical compounds or even for hemoperfusion [37].

**Figure 2:**
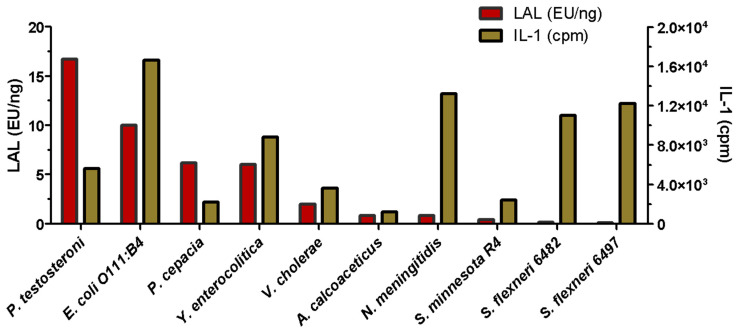
Comparison of LAL and IL-1 activities of LPSs extracted from different bacterial genera. (Adapted from Laude-Sharp et al., 1990 [40]).

Difficulties in detecting LPSs in human fluids were identified very early on. The Limulus Amoebocyte Lysate or LAL method, described below, notably failed to detect the passage of LPS molecules through the membranes in dialysis tanks used to filter the blood of patients with kidney failure [38]. Two major problems were highlighted: the difficulty of testing LPS in blood, and the fact that LPS molecules, when crossing the dialysis membrane, were in dissociated forms and not recognized in the way LPS aggregates can be detected. Fortunately, IL-1 cytokine induction, which is responsible for fever [39], was detected and quantified. After bacterial characterization of two *Pseudomonas* strains isolated from the dialysis baths, the corresponding LPSs were extracted and labeled with tritium. These labeled LPSs were used, on small reconstituted dialysis devices, to demonstrate that different *Pseudomonas* LPSs were able to cross the three different membranes used in hospitals at that time [38]. Following this discovery, different LPSs were further tested and compared for their IL-1-inducing capacities compared to the LAL detection level. The diagram presented in Figure 2, adapted from [40], shows the variability of the responses and the absence of a correlation between LAL and the IL-1 cytokine responses.

These results are not surprising since these two methods were found to detect different LPS structures, as well as their different aggregation states [41,42]. Such discrepancy was a major discovery in the domain, and kidney dialysis membranes were improved following this work. It is important to remember that at this time, LPS molecules were not yet amenable to Mass Spectrometry, and the molecular weights were therefore poorly estimated. As LPSs formed large aggregates, MWs were even more over-estimated. We solved this issue by obtaining the first native LPS mass spectra [43]. The kidney dialysis LPS contamination experiments clearly demonstrated that in such critical cases, the simultaneous use of two different LPS detection methods is a safe solution.

Determination of endotoxin activity is a major concern for pharmaceutical industries [44]. All drugs or medical devices must comply with an endotoxin limit. The world’s leading pharmacopeias have agreed to authorize two techniques for endotoxin determination: the rabbit pyrogen test (RPT) [45] and the Limulus Amoebocyte Lysate (LAL) [46].

The RPT consists of injecting intravenously a solution of LPS into rabbits and monitoring changes in body temperature. If a 0.6 °C increase in the animal body temperature is observed upon intravenous injection, and if at the same time the sum of temperature increases in three rabbits exceeds 1.15 °C, this invalidates the use of the solution. This method is very sensitive, and the rabbit model has been shown to be the closest species to humans in endotoxin response. However, non-animal tests will soon replace this test [47].

The LAL is a highly sensitive test based on an enzymatic cascade found in the lymph of the horseshoe crab *Limulus* species [46]. When LPSs are combined with this enzymatic cascade, the blood clots, proportionally to the amount of each given LPS. Recently, the European Pharmacopoeia authorized two additional techniques for determining LPS activity: the recombinant factor C (rFC), a recombinant protein from the LAL enzymatic cascade, and the monocyte activation test (MAT) [48]. In MAT assays, monocytes are activated by endotoxins and the release of the pro-inflammatory interleukins is assayed by ELISA [49]. All these techniques aim to determine the endotoxin activity and do not only reflect the quantity of LPS molecules. Except for RPT, the results are expressed in Endotoxin Units (EUs) or their equivalences (EEU). This EU is determined using a reference endotoxin standard known as RSE (Reference Standard Endotoxin). Originally, 1 EU was equivalent to 0.1 ng of an endotoxic LPS from *E. coli* O113:H10:K- [50], but nowadays, the more recent available RSE batch displays 1 EU = 0.12 ng [51]. Of course, due to the major diversity in LPS structures, it is difficult to rely on a single standard. This point will be illustrated in the present work.

Although they are not recognized by the Pharmacopoeia, other techniques have been developed to determine LPS levels. An in vitro kit is commercially available using the engineered human embryonic kidney HEK293 cell line reporter cells expressing the human or mouse TLR-4 receptor and its co-receptors (MD2/CD14). This in vitro assay enables the evaluation of the TLR-4 dependent NF-κB response upon stimulation with LPSs [52]. ELISA kits are available but are poorly specific, as there are no antibodies capable of recognizing all lipopolysaccharides [53]. 

In the present study, we analyzed various well-characterized and highly purified LPSs presenting diverse structures using four different LPS detection methods including LAL, HEK-blue TLR-4, LC-MS^2^, and MALDI-MS [54], the latter requiring more material than the three others. We showed that the different LPS detection methods respond differently to their extensive structural diversity, impacting the immune system through different pathways. This diversity explains why LPS structures, unlike other classes of molecules, cannot be measured and estimated using a single technique. 

## 2. Results

### 2.1. Illustration of LPSs’ Diversity by SDS-PAGE Electrophoresis of the Different LPS Samples Used in This Work

**Figure 3:**
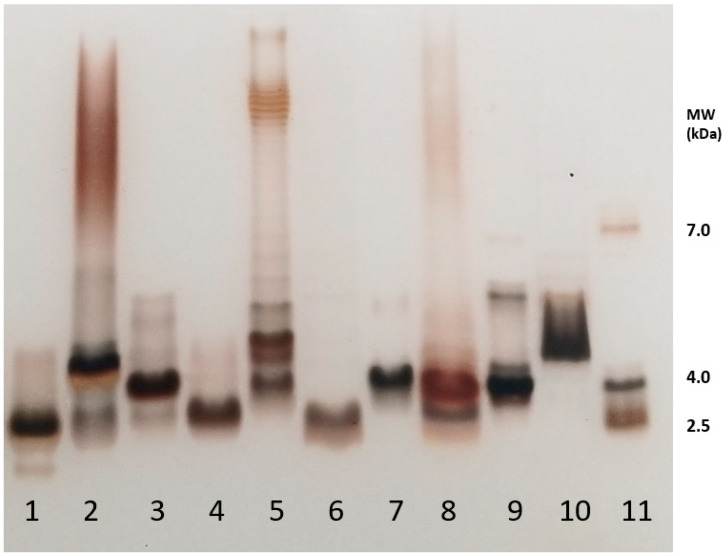
SDS-PAGE analysis of the different LPSs used in the present study. 1—*Y. pestis*; 2—*Y. enterocolitica*; 3—*B. pertussis* 1414; 4—*B. pertussis* A100; 5—*E. coli* 0119; 6—*E. coli* J5; 7—*N. meningitidis*; 8—*P. aeruginosa* P3; 9—*S.* Typhimurium Ra; 10—*R. pickettii*; 11—LPS-MW Standard. Staining was performed according to Tsai and Frash [55]. Rough-type LPSs migrate in the low-masses level at about 4 kDa.

SDS-PAGE is often the first tool used to characterize and compare LPS molecules as it gives precious and quick data on their size and heterogeneity. This method also allows for classifying the given LPS samples into one of the three categories, i.e., Rough-, Semi-Rough-, or Smooth-type LPSs (Figure 1). Smooth-type LPSs display lipid A, a core, and O-chains; Semi-Rough LPSs display lipid A plus a core plus a single O-chain unit; and Rough-type LPSs display lipid A plus a core. SDS-PAGE profiles are also informative on their global purity, heterogeneity, and approximative molecular masses. LPS profiles of *Yersinia pestis*, *Y. enterocolitica* O3, *Bordetella pertussis* 1414 LOS and A100 mutant LPS, *Escherichia coli* O119, *E. coli* J5, *Neisseria meningitidis*, *Pseudomonas aeruginosa*, *Salmonella enterica* Typhimurium Ra type, and *Ralstonia pickettii* were compared here by this technique (Figure 3, lanes 1–10). They were tested in parallel to a laboratory-made standard mixture of three different LPSs (Figure 3, lane 11) with MW previously measured by mass spectrometry.

*Y. pestis* LPSs isolated from bacteria grown at 37 °C (line 1) correspond to Rough-type LPSs migrating deeply to the level of low-molecular-weight molecules. *Yersinia enterocolitica* O3 LPSs from bacteria grown at 37 °C (line 2) correspond to Smooth-type LPSs with some heterogeneity illustrated by the presence of four major molecular species in the lower mass region and a smear at the level of LPSs containing O-chains. LOSs of *Bordetella pertussis* strains Tohama (line 3) with a main molecular dodecasaccharide core species migrate less than *B. pertussis* A100 LOS (line 4), with a shorter nonasaccharide. The difference between these two LOSs is the absence of the terminal immunogenic trisaccharide in *B. pertussis* A100. The *E. coli* O119 4184 strain LPS (line 5) displays a typical Smooth-type profile with high-molecular-weight species organized in a ladder-like pattern. Some short LPS molecules without any O-chain appear in the low mass region [56]. The *E. coli* J5 (Rc mutant) short LPS displays a characteristic profile of Rough-type molecular species with shortened cores (line 6). The *Neisseria meningitidis* LOS being known for absence of O-chains gave, as expected, a single-band LOS profile (line 7) at about 4 kDa. *Pseudomonas aeruginosa* LPS (line 8) displays a characteristic profile of a Smooth-type LPS with a faint higher molecular-weight region and short-core molecular species without O-antigens. In the higher mass region, two subregions could be distinguished with the A-band O-antigen, and the B-band O-antigen LPS migrating in the middle [57,58]. *Salmonella* Typhimurium Rough-type Ra LPSs appear, as expected, in the low-molecular-weight region (line 9). *Ralstonia pickettii* [59] LPSs’ migration profile (line 10) was previously described [60], and it corresponds to a high-molecular-weight molecular species of short LPSs. This LPS migrated in the low-mass region with molecular species corresponding to LOSs and LPSs up to four O-chain units. On the right side of the gel (line 11) is the in-lab standard corresponding to LPS molecular species of 2 kDa, 4 kDa, and 7 kDa, respectively, as characterized by MALDI mass spectrometry. Such an LPS standard gives more accurate information on the different LPS molecular masses than the often-used protein standards. Standard proteins migrate at much higher levels leading to the over-estimation of LPS molecular weights and thus major errors.

A comparison of the profiles of the samples used here illustrates LPS structural diversity. It also gives major information on the polysaccharide length and heterogeneity of these molecules with very little quantities (0.1 to 0.4 µg).

### 2.2. Impact of Growth Medium Temperature Conditions on Two Yersinia Species

Earlier, we described for the first time the structure of *Y. pestis* lipid A. We also reported some *Yersinia enterocolitica* lipid A structures when grown at 37 °C and those of other *Yersinia* species, i.e., *Y. pestis*, *Y. ruckeri*, and *Y. pseudotuberculosis* [61]. All were different, and in contrast to a previous claim [62], their lipid A structures differed from *E. coli* lipid A. In fact, although the molecular weights of both hexa-acyl lipid As (1798 u) were the same for both genera, different FA distributions accounted for this confusion. *Yersinia enterocolitica* O3 displays a branched FA at C2′ with 14 carbons compared to 12 carbons in *E. coli*. Both lipid As also differed by the nature of a branched FA at C3′ being 12:0 for *Yersinia* and 14:0 for *E. coli* resulting in the same global (C2′ + C3′) MW. 

As *Y. pestis* was found not to be virulent when grown at 27 °C, the first structure we described in 2000 corresponded to bacteria cultivated under these safe conditions. We can see here, as confirmed by other authors [63,64,65], that unsaturated 16:1 FAs are present at low temperatures and absent at 37 °C. In the present work, we compare LPS structures by MALDI-MS to the results obtained by LAL, HEK-blue hTLR-4, and LC-MS^2^ for this species grown at 27 °C versus 37 °C.

#### 2.2.1. Impact of Growth Temperature on *Yersinia pestis* LPSs

##### Impact on *Y. pestis* Lipid A Structures

**Figure 4:**
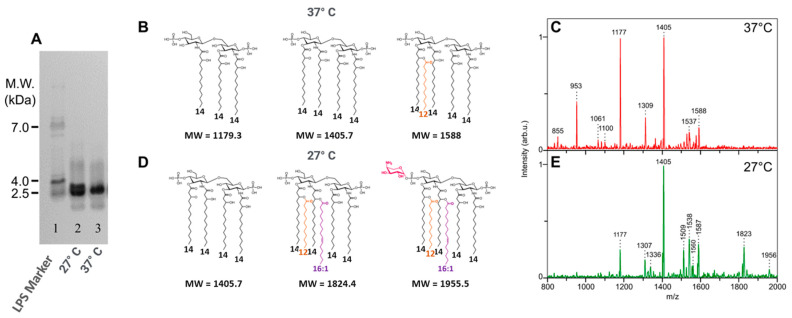
Comparison of *Y. pestis* grown at 27 °C vs. 37 °C: (**A**) comparison of LPS profiles on SDS-PAGE, 27 °C (line 2) and 37 °C (line 3). Comparison of lipid A structures ((**B**) 37 °C; (**D**) 27 °C) and MALDI mass spectra ((**C**) 37 °C, (**E**) 27 °C). LPS molecular weight marker (line 1). Fatty acids other than 14:0-3-OH are presented with different colors.

Little differences were found on SDS-PAGE electrophoresis when *Y. pestis* LPSs were compared (Figure 4A), although at 27 °C, a double band appears, compared to the LPS profile at 37 °C.

The comparison of both lipid A structures by MALDI-MS (Figure 4C,E) shows important differences. At 37 °C, the main molecular species appears in the lower-mass region of the spectrum and corresponds to a major tetra-acyl molecular species signal at *m*/*z* 1405 corresponding to the antagonist tetra-acyl lipid A form [66,67], and it follows a tri-acyl molecular species at *m*/*z* 1177 and a di-acyl molecular species at *m*/*z* 953. Although present in small amounts, the highest masses correspond to a penta-acyl molecular species (Figure 4B).

When grown at 27 °C, the temperature of the plague vectors’ bodies (fleas), the antagonist tetra-acyl molecular species is again the more important one, at *m*/*z* 1405 (Figure 4D). A few penta-acyl molecular species appear at *m*/*z* 1587 and 1560, followed by a hexa-acyl molecular species at *m*/*z* 1825 containing an unsaturated 16:1 fatty acid as confirmed by GC-MS data [61]. A small molecular species at *m*/*z* 1956 corresponds to the substitution of one phosphate group with AraN (Figure 4D).

Slight differences were observed in the core moieties confirming the differences seen on the SDS-PAGE profiles (Figure 4A).

##### Impact of Growth Temperatures on *Y. pestis* LPS Detection

**Figure 5:**
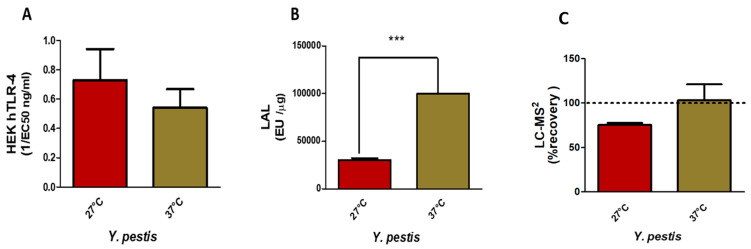
Comparison of HEK hTLR-4 (**A**) and LAL (**B**) activities and LC-MS^2^ (**C**) quantification of *Y. pestis* LPS from bacteria grown at 27 °C versus 37 °C (*** *p*-value < 0.0001).

Results of HEK-blue hTLR-4 (Figure 5A) assays indicated a low response for both temperatures, which can be explained by the presence of major tetra-acyl antagonistic molecular species. These LPSs are therefore much less toxic. The slight differences for *Y. pestis* grown at 27 °C can be explained by the presence of AraN and a few hexa-molecular species at *m*/*z* 1825 with the presence of an extra 16:1 FA. This peculiarity results in a low inflammatory response, hence the low values detected in the assay.

LAL activities (Figure 5B), on the contrary, are significant, but as both LPSs are not endotoxic, here again, LAL detection is not correlated with toxicity but rather to the presence of LPS molecules.

LC-MS^2^ analyses (Figure 5C) gave 75 % recovery for *Y. pestis* grown at 27 °C and 93 % for *Y. pestis* grown at 37 °C, both values being coherent with what was expected for these samples and confirmed by MALDI-MS.

#### 2.2.2. Impact of Growth Temperatures on *Yersinia enterocolitica* LPSs

##### Impact on *Y. enterocolitica* Lipid A Structures

**Figure 6:**
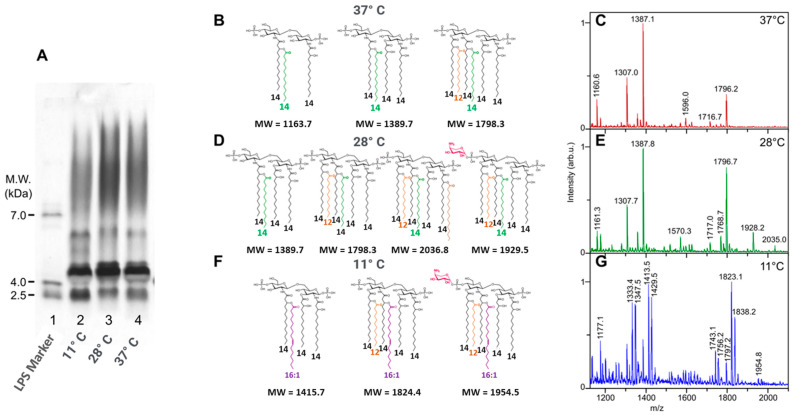
Comparison on SDS-PAGE of *Y. enterocolitica* grown at 11 °C, 28 °C, and 37 °C ((**A**) 2,3,4). Comparison of lipid A structures of *Y. enterocolitica* grown at 37 °C, 28 °C, and 11 °C (**B**,**D**,**F**), and MALDI mass spectra of LPSs from *Y. enterocolitica* grown at 37 °C, 28 °C, and 11 °C (**C**,**E**,**G**). LPS molecular weight marker (line 1). Fatty acids other than 14:0-3-OH are presented with different colors.

Here, we describe the differences observed for LPSs on SDS-PAGE (Figure 6A) and in MALDI-MS on the lipid A moieties isolated from the same *Y. enterocolitica* strain, grown at three different temperatures (Figure 6C,E,G: 37 °C, 28 °C, and 11 °C). 

*Yersinia enterocolitica* O3 grown at 11 °C (Figure 6G) gave an LPS profile different from those of the same bacteria grown at 28 °C (Figure 6E) and 37 °C (Figure 6C). LPSs obtained from bacteria grown at 11 °C displayed fewer O-chains compared to the two others (SDS-PAGE profiles Figure 6A). Another difference appears in the lower molecular mass region with the fastest migrating band being more intense at 11 °C than at the two other temperatures (Figure 6A, lane 2 (11 °C), lane 3 (28 °C), and lane 4 (37 °C)). 

It is interesting to note that, thanks to the MALDI-MS of lipid As and GC-MS of the FAs, we found that in *Y. enterocolitica*, the unsaturated 16:1 FA is not incorporated at 27 °C, as it was in *Y. pestis*, but appears at much lower temperatures, of 11 °C. These results are different from *Yersinia pestis* in which these fatty acids already appeared at 28 °C [61]. 

As shown in Figure 6, when bacteria are grown at 37 °C, the higher molecular species signals are observed at *m*/*z* 1796-7, corresponding to a hexa-acyl molecular species. At 28 °C, an additional hexadecenoic acid is added at the branched C-2 position on the amide-linked FA on GlcN-1, to give a hepta-acyl molecular species at *m*/*z* 2036, while an AraN residue is added to the phosphate group of GlcN-2, accounting for the origin of the signal at *m*/*z* 1929. Both modifications are usually known to appear under stress conditions [68]. When bacteria were grown at 11 °C, the corresponding lipid A structures contained 16:1 unsaturated FA signaling at *m*/*z* 1823 like the one observed in *Y. pestis* grown at 28 °C.

##### Impact on *Y. enterocolitica* LPS Detection

These different temperature variants of *Yersinia enterocolitica* O3 LPS, presenting structural differences characterized in their lipid A moieties, were compared by LC-MS^2^, LAL, and HEK-blue TLR-4 tests, and all were found to be weak inducers. Lipid A structure at 28 °C, compared to 37 and 11 °C, differed mainly by the presence of AraN. As shown in lipid A spectra in Figure 6, the impact of this AraN derivative is significant on the HEK-blue TLR-4 activity signaling through NF-κB.

**Figure 7:**
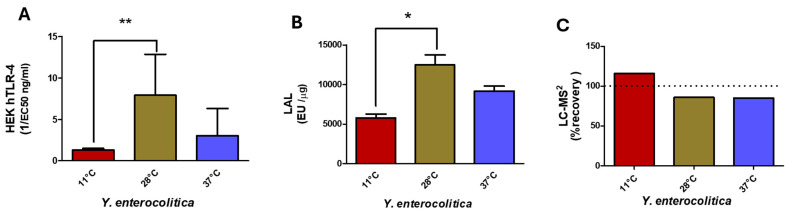
Comparison of HEK hTLR-4 (**A**) and LAL (**B**) activities, and LC-MS^2^ (**C**) for *Y. enterocolitica* LPS from bacteria grown at 11 °C, 27 °C, and 37 °C. (* *p*-value < 0.01; ** *p*-value < 0.001).

Results of the HEK-blue hTLR-4 assays (Figure 7A) indicated that LPSs present in bacteria grown at 11 °C gave the lowest response in this assay. This can be easily explained by the presence of the 16:1 FA, not only because the length of this FA is known to hinder a good fit in the TLR4-MD2 pocket but also because the presence of the unsaturated bond increases this effect. LPSs from the 28 °C culture recorded the highest activity in the HEK-blue hTLR-4 assay compared to both others. This can be explained by an increased amount of hexa acyl lipid A and the presence of AraN on the phosphate group.

The three *Yersinia* temperature variant LPS structures showed variable toxicity in the LAL assay (Figure 7B). All displayed high values, even if all samples contained high amounts of the non-toxic tetra-acyl molecular species. Furthermore, the sample at 11 °C should not be toxic due to the addition of an unsaturated 16:1 as shown by weak HEK-blue hTLR-4 responses. 

LC-MS^2^ analysis (Figure 7C) gave 115%, 86%, and 85% recovery for *Y. enterocolitica* grown at 11 °C, 28 °C, and 37 °C, respectively, all values being coherent with what was expected for these samples and confirmed by MALDI-MS.

### 2.3. Impact of Lipid A Phosphate Group Decorations and Fatty Acid Number on LPS Biological Activities and Detection

#### 2.3.1. Comparison of TLR-4 Induction and LAL Detection with Tetra- and Hexa-Acylated Lipid A in *E. coli* J5 LPS

**Figure 8:**
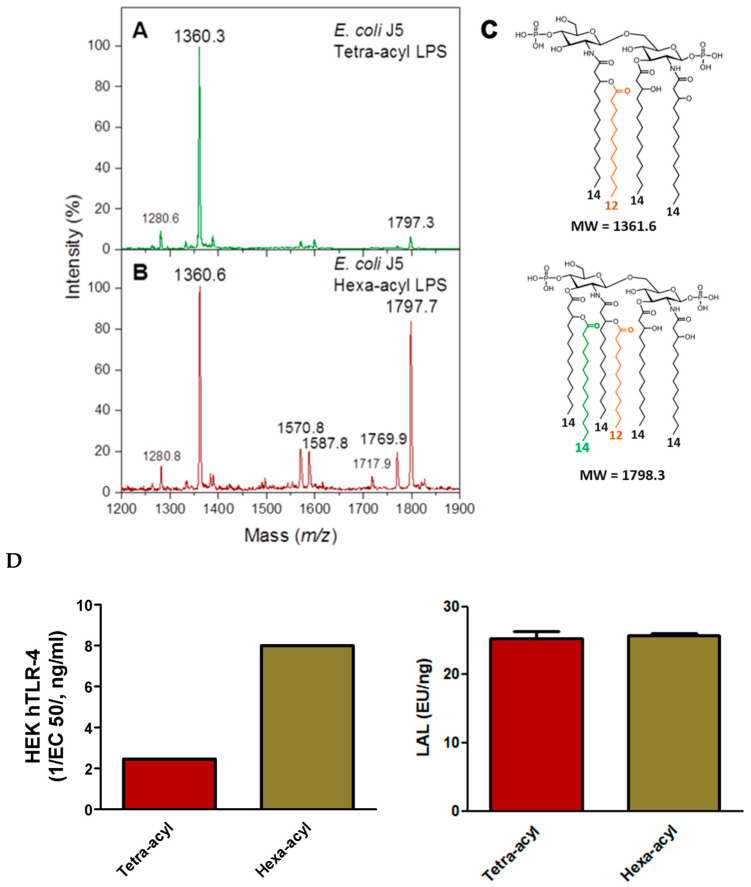
MALDI mass spectra obtained for tetra-acyl *E. coli* J5 lipid A (**A**) and hexa-acyl *E. coli* J5 lipid A (**B**). Corresponding lipid A structures (**C**). Comparison of LAL and HEK-blue hTLR-4 activities (**D**). Fatty acids other than 14:0-3-OH are presented with different colors.

Two *E. coli* J5 LPSs, isolated from the same strain and differing only by the number of FAs present in their lipid A moieties, were compared by MALDI-MS analysis as described in the Methods section (Figure 8A–C). The two LPSs were then compared for LAL detection and HEK-blue hTLR4 activities. The hexa-acyl LPS corresponded to the classical *E. coli* lipid A structure, known as being a highly inflammatory molecule compared to the tetra-acyl LPS characterized by the absence of the two branched 14:0-3-OH and 14:0 FAs at position C3′ on GlcN2.

As expected, the hexa-acyl LPS, although containing some tetra-acyl molecular species, was much more active in HEK-blue hTLR-4 assays than the major tetra-acyl LPS (Figure 8D). However, no differences were observed between these two LPSs in the LAL assay, illustrating that LAL is efficient in detecting both LPSs but is not indicative of their toxicity. The LAL assay is very sensitive to the presence of the two phosphate groups in the lipid A structure known to be important for agonist properties.

Both LPSs were correctly detected by the LC-MS^2^ method, as expected being based on the presence of the 3-OH FA markers, and it detected three 14:0-3-OH versus four 14:0-3-OH per molecule for the tetra-acyl versus hexa-acyl LPS.

#### 2.3.2. Comparison of TLR4, LAL, and LC-MS^2^ Analyses in LPSs from Different Genera with Various Lipid A Structures

Different LOSs, originating from different genera and characterized by our group [69] were selected to illustrate the impact of phosphate decorations, FA numbers, and carbon chain lengths on the three selected detection methods. For optimal comparison by avoiding discrepancies due to solubility or MWs, all selected LPSs were Rough-type LPSs or LOSs with similar molecular weights. It is important to note that the biologically active lipid A moiety represents about 50% of Rough-type LPSs, while it decreases to 20% in Smooth-type LPSs. However, this is seldom taken into account as masses are mostly used in biological activities and not molar amounts.

**Figure 9:**
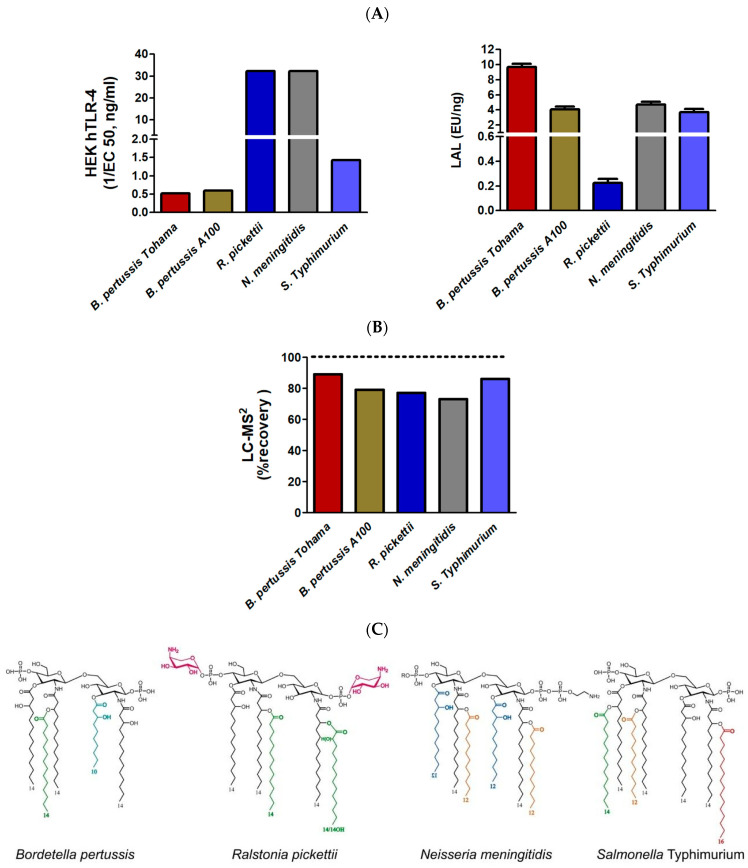
Comparison of the LAL and HEK-blue TLR-4 activities (**A**) and LC-MS^2^ quantification (**B**). Corresponding lipid A structures (**C**) obtained from *B. pertussis* 1414 and A100 strains, *Ralstonia pickettii*, *Neisseria meningitidis*, and *Salmonella* Typhimurium Ra. Fatty acids other than 14:0-3-OH are presented with different colors.

Figure 9A compares responses of the five LOS samples to hTLR4 HEK-Blue and LAL tests. We can see that these selected tests respond differently to different samples, and there is no correlation between the two methods. The response variations of the hTLR4 HEK-Blue test can be easily explained by referring to the structures depicted in Figure 9C. The highest response corresponds to LOSs from *Ralstonia picketti* and *Neisseria meningitidis*. *N. meningitidis* lipid A is hexa-acylated with FA chains containing 12 to 14 carbon atoms. This is the most active combination. Moreover, substitution of the phosphate group at the C1 position with Phospho-ethanolamine residue also contributes to enhancing the TLR4 activation. Lipid A from *R. picketti* is penta-acylated; hence, some decrease in TLR4 activation could be expected if both phosphate groups were not substituted with Aminoarabinose residues, powerful structural determinants of the TLR4 activation. As for the two LOSs from *B. pertussis* strains, they respond weakly because their lipid A is under-acylated and the phosphate groups are not substituted in the tested mutants. As for the *S.* Typhimurium Rough-type LPS, its hepta-acyl lipid A is over-acylated and phosphate substitutions are missing. This is why its response is also weak. 

As for the LAL test, four of the five samples respond similarly while the two *B. pertussis* samples are very weak inflammatory inducers compared to the others. The only exception is the LOS from *R. picketti* whose response is at least an order of magnitude lower than those of other samples, while it is the highest inflammatory LPS in this series. In this example, again LAL response and toxicity are not in adequation. Considering lipid A structures, we can speculate this is due to the efficient neutralization of negative charges of the phosphate groups first by esterification, second by positive charges of the AraN residues.

The HEK-Blue TLR-4 responses obtained with the five different selected LPSs, including a second *B. pertussis* strain, corresponded to what was expected from their structure. 

In the same way, LC-MS^2^ gave coherent responses (Figure 9B), between 70% and 90% of that which was expected by calculation of the corresponding theoretical values. LAL detection was not coherent in the five examples and could not be related to endotoxicity.

## 3. Discussion

Endotoxin detection is a difficult task due to the multiple LPS structures elaborated by Gram-negative bacteria during their biosynthesis, and to the extensive diversity of their O-chains [70,71].

**Figure 10:**
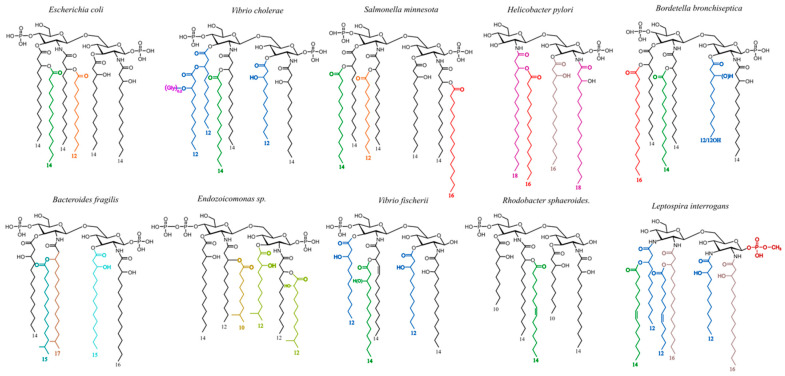
Examples of lipid A structural diversity in different bacterial genera. Fatty acids other than 14:0-3-OH are presented with different colors.

Regarding lipid As, it has been long thought, and still mentioned in the literature, that the lipid A structures of LPSs were common to all the species in a given genus. This holds true mainly for the *Escherichia* genus and Enterobacteria which are the most extensively studied. In the 1990s, we showed that many other bacterial genera, like the *Bordetella* genus [72], *Helicobacter*, and *Yersinia* [61], display high diversity in their lipid A structures despite sharing some common traits. Part of this diversity is illustrated in Figure 10 where different colors are attributed to different FAs and where the amino sugar backbones also display some differences in nature and substitutions.

The more common lipid A structures are made of phosphorylated glucosamine (GlcN) disaccharide substituted with four to six 10- to 16-carbon fatty acids (FAs), among which include mainly 3-OH (3-hydroxy) FA [73]. Many other lipid A structures can be found with a di-amino-di-GlcN disaccharide backbone [74], phosphorylated or not. As for the FA, their number can increase up to nine and can have up to 28 carbons atoms or even more [75].

Many lipid A structures, different from the canonical hexa-acyl and bis-phosphorylated *E. coli* lipid A, have been described during the last decades. Some are known for weak cytokine-inducing capacities, helping the bacteria bearing them to evade the host immune system [76]. A good example is *Helicobacter pylori* lipid A containing only a few FAs but with long carbon chains and displaying a single phosphate group on the glucosamine disaccharide. This lipid A is a poor stimulator of the TLR-4/MD2 complex, allowing the bacteria to evade the host immune response and establishing well-known long-term colonization at the level of the gastric epithelium [77,78,79,80,81]. This weak activation partly explains why these bacteria are responsible for infections in as much as about 50% of the world population [82]. However, the nature of their O-chains, mimicking blood group oligosaccharide epitopes, certainly participates in the absence of their rejection [83,84].

Structural diversity is further enhanced by structural modifications of the LPS molecules after their export into the external membrane [85]. Bacterial detection and evasion involving LPS recognition can be significantly impacted by late-stage biosynthesis steps, like the addition of sugars and FAs taken from membrane phospholipids. Other structural elements like ethanolamine phosphate or amino acids originate from other elements constituting the membrane. An important factor impacting LPS structure is the adaptation of bacteria to growth conditions such as temperature, medium, or environmental stress [65,84,86]. Therefore, bacteria growing in the natural environment, or in the intestinal tract with strong growth condition differences, harbor different LPS structures.

A strong impact of the addition of 16:0, on GlcN-1 at the secondary C2 position, to *E. coli* and *Salmonella* lipid A structures [87], among others, was demonstrated with different strains and species. For example, with *E. coli* grown in Biofilms, the addition of a 16:0 FA was observed [88]. Lipid A palmitoylation does not influence bacterial adhesion, but it diminishes the inflammatory response and enhances resistance to some antimicrobial peptides. Palmitoylation of lipid A protects bacteria from the host TLR4-dependent immune defenses, reducing the detection efficacy of such molecular species [89,90]. However, palmitoylation is rarely complete so that HEK-blue TLR-4 can still detect the inflammatory LPS forms.

We previously showed that *E. coli* lipid A palmitoylation increased the in vivo survival of biofilm bacteria in a clinical model of catheter infection, potentially contributing to biofilm tolerance to host-immune defenses [88]. 

This was also observed in the present work with the diminished inflammatory activity of the *Salmonella* Typhimurium Ra LPS harboring a branched 16:0 substitution, reducing the inflammatory activity of this LPS, as observed in HEK-blue TLR-4 activation assays.

Other “decorations” to LPS structures greatly impact their biological activities and LPS detection. Some *B. pertussis* strains have free additional GlcN residues on their lipid A phosphate groups that strongly enhance TLR-4 activation. Their presence was also observed on *B. avium* lipid A, in contrast to *B. trematum*, and *B. hinzii*, both of which display the same FA distribution [72].

When lipid A structures are not well recognized by the TLR-4 receptor, bacterial virulence is enhanced as shown under biofilm growth conditions. Another good example of a lipid A structural modification that enables the bacterium to escape the human immune system is that of *Yersinia pestis*, the plague agent [61]. This structure was impacted by the body temperature of the flea vector, leading to the incorporation of an unsaturated 16:1 residue. This lipid A also contained a large amount of tetra-acyl molecular species (Figure 3) which are known to induce a weak inflammatory response. Such modifications of the FA chain length and hypo-acylation led to a structure not detected by the host-immune defense system, allowing such bacteria to develop in the host. 

In the present work, we modified the growth temperature conditions of another *Yersinia* species, *Y. enterocolitica*, to observe and compare the impact of temperature on the lipid A structures. The modifications were different from those observed with *Y. pestis* at the tested temperatures. Interestingly, in this species, decreasing the growth temperature to 28 °C did not lead to the addition of an unsaturated 16:1-3-OH at GlcN-2 as in *Y. pestis*. Such modification that enhances the fluidity of the external membrane in response to cold temperatures was, however, observed at the lower temperature of 11 °C. The number of FAs was not diminished, and very little 16:0 was added to GlcN-1. These more discrete changes did not significantly impact the HEK-Blue TLR-4 recognition, but LAL was reduced by 50% (Figure 7).

It has been shown that lipid As with six FA chains display optimal inflammatory activity [30], while those with five FA chains are about 100-fold less active, and those with four FA chains lack any agonistic activity. Based on crystal structure, the authors were able to predict that in LPSs with less than six FA chains, the lipid A molecule moves deeper into the MD2-TLR-4 pocket, and consequently MD-2 does not dimerize with TLR-4 to induce the cytokine production. 

We showed in this work that important structural modifications of *E. coli* J5 lipid A structure [91] did not change its capacity to activate the LAL assay but impacted the inflammatory response in the HEK-Blue hTLR-4 test [92]. The hexa-acylated form of this LPS results in TLR4 activation capacities reduced three-fold compared to its tetra-acylated form, which is expected [93] due to the mentioned reduced activation of under-acylated lipid As (Figure 8D). By comparison, no modification was observed when the LAL test was applied when comparing the tetra and hexa-acyl lipid A forms. This confirms that the LAL method, known for its good sensitivity, was unable to discriminate between inflammatory and non-inflammatory LPS molecules, and it is therefore not sufficient to define levels of LPS toxicity (Figure 8D). 

Other structural details concerning FA nature are known for impacting the LPS inflammatory induction capacities. *B. pertussis* LPS is well known for its weak inducing capacities, notably because this LPS is under-acylated with fewer FAs (at most five FA), and, depending on the strain, with the presence of one or two short-chain FAs 10:0-3-OH [64].

We showed here that two strains of *B. pertussis*, Tohama and A100 [15], did not induce much HEK-Blue hTLR-4 response, illustrating their low toxic capacity levels, as expected, while they induced a LAL response similar to other well-known toxic LPSs such as those of *Neisseria* (Figure 9). In this work, the *Salmonella* Typhimurium Ra-type LPS was also tested and as the lipid A was carrying a branched 16:0 FA at C-2 of GlcN-1, we observed an expected low level of activation in the HEK-Blue hTLR-4 test. Here again, the LAL test gave similar responses, both for the highly inflammatory *N. meningitidis* LOS and for the weak LOS inducer *S*. Typhimurium.

Other interesting structural details impacting the inflammatory capacities of LPS/LOS are the decorations present on lipid A phosphate groups, like PEA for *Neisseria* or *E. coli*, AraN for *E. coli* or *Ralstonia* [60], and GlcN for *Bordetella* [64]. The LPS of *Ralstonia pickettii* tested in this work carries AraN on both phosphate groups which explains its very weak detection by the LAL test and TLR-4 activation as strong as that of *N. meningitidis* LPS. Earlier, we also demonstrated that LPSs with both fewer FAs and AraN substituents resulted in lower cytokine-inducing capacities [60,94]. 

The data obtained in this work show clearly that no single method for LPS detection and quantitation can be satisfactory. We consider below five major methods used for endotoxin detection and quantification, i.e., RPT, LAL, MAT, TLR4-HEKblue, and LC-MS^2^ quantification of 3-hydroxy FA.

For ethical reasons, the in vivo rabbit pyrogenic test (RPT) will disappear in favor of in vitro tests [47]. However, all techniques developed to assay endotoxins and replace this test have their benefits and disadvantages.

LAL is a very sensitive method with a LOD of 0.005 EU/mL (or 0.6 pg/mL). However, LAL does not distinguish between endotoxic active and antagonist LPS. One major problem is that *E. coli* LPS used as the LAL standard cannot represent all LPS structures. Use of the real corresponding LPS reference, when identified and available, in addition to the regular *E. coli* one would provide more relevant values. Moreover, LAL is based on an enzymatic cascade in which the proteins are subject to various physicochemical parameters. Temperature, pH, and ions can directly inhibit the LAL assay [95]. Some molecules, such as polysaccharides, are also known to interfere with the LAL test [96,97]. Complex matrices, such as blood, saliva, or other biological fluids can complicate the assay [98,99]. Some factors can also disrupt LAL analyses over time. The best-known phenomenon is the Low Endotoxin Recovery (LER), a masking effect due to the formation of a complex blocking the ability of Factor C, the main component in LAL detection. A mixture of citrate, polysorbate, and saline buffer (commonly found in pharmaceutical ingredients) always causes such an LER effect [100,101].

The MAT has the advantage of using human monocytes, but most often, cell lines limit the number of cytokines detected. However, this technique is probably the best method for anticipating the behavior of circulating LPSs, but interleukin titration by the MAT assay is not endotoxin-specific. The presence of contaminants such as nucleic acids or lipopeptides may induce the production of pro-inflammatory cytokines and chemokines. The use of cells expressing only TLR4 receptors confers high endotoxin specificity upon the test. But in both cases, the use of living cells makes this methodology sensitive to many external parameters. The sensitivity remains very interesting with a LOD close to 0.05 EU/mL (6 pg/mL). Unfortunately, the MAT test remains expensive and mainly relies on one cytokine measurement, so we selected HEK-Blue™-hTLR4 assay for this study as it is more accessible to users. Like for MAT, this test uses human cells and is likely to produce a close-to-reality response. In fact, MAT detects the production of cytokines while the HEK-Blue™-hTLR4 assay detects the activation of the NF-κB pathway. MAT is, however, less sensitive than the rFC [102]. 

The LC-MS^2^ quantification of specific markers is a robust method, and being a chemical method, it works well for all LPS types. Although less sensitive than LAL or MAT, with a LOD of 12 pg/mL, the huge advantage is the limited impact of external interferences. Solubility, pH, temperature, or salinity do not interfere with such quantification, mainly because difficult conditions are required to prepare the markers. For example, strong hydrolysis with HCl is necessary to liberate the hydroxy fatty acids [103]. 

LPSs also contain rare molecules that can be targeted for quantification. Kdo or heptoses are often described as specific markers. These carbohydrates or lipids can be quantified using traditional biochemical techniques [104,105]. Gas chromatography or LC-MS^2^ are also used to quantify monosaccharides and fatty acids levels [106,107]. It is also important to note that 3-OH FAs are the best LPS markers, because 3-deoxy-D-manno-2-octulosonic acid, Kdo [108,109], and Heptose [110] have also already been described as being present in other bacterial components such as capsular PS. In addition, when Kdo is phosphorylated in position 4 by a phosphate group, like in *Bordetella*, *Vibrio cholerae*, *Haemophilus*, and *Actinobacillus*, the molecule is modified upon hydrolysis and becomes non-detectable, if not treated with hydrofluoric acid before quantification [108].

LPS structural details exert strong influence on LPS toxicity as shown by cytokine induction and MAT or HEK-Blue hTLR-4 signalization, but LAL is not sensitive to these modifications and again did not differentiate the toxic and non-toxic LPS forms [111]. Nevertheless, LAL is highly sensitive, but it should be complemented by other methods like the HEK-Blue hTLR-4 signal or MAT assays or LC-MS^2^ for defining LPS toxicity.

In addition to the fact that some LPSs are naturally not toxic, chemical modifications of LPS structures have been used for decades to separate the beneficial, from the deleterious, activities of these molecules. In the early 1980s, we demonstrated that dephosphorylation of the *Bordetella pertussis* lipid A moiety created a non-toxic, non-pyrogenic molecule that retained adjuvant properties but without high inflammatory properties. We recently pursued such experiments to obtain more powerful and beneficial molecules by maintaining the oligosaccharide moiety linked to lipid A during the liberation of its glycosidic phosphate. The complete molecule is much more active than the isolated non-natural mono-phosphorylated lipid A and other natural or synthetic MPLs (MPL and GLA). This is explained by the fact that the LPS molecule in its entirety retains more of the native molecule conformation recognized by the TLR-4 receptor than lipid A alone. The latter is also an artifact produced by acid hydrolysis, even though the lipid A moiety is known as the most active part of LPSs. The whole LPS conformation and its physicochemical properties are important for its recognition and its activity. Therefore, LPS toxicity can be mastered by chemical modifications, as we recently demonstrated in oncology models, with chemically detoxified LPS used as immunomodulators in immunotherapy [112].

## 4. Material and Methods

### 4.1. Bacteria

*Bordetella pertussis* and *Neisseria meningitidis* growth batches were kind gifts of the Institut Mérieux, Lyon, France.

Smooth-type strains of *Y. enterocolitica* O:3 as well as *E. coli* O119 strain 4184, from the National Research Council (NRC) collection of Canada, were kind gifts from the late Dr. M.B. Perry. *Yersinia* were grown as described at different temperatures, 37 °C, 28 °C, and 11 °C, in previously described conditions [113].

*Escherichia* growth conditions: Biomass was obtained based on the protocol described in [56].

*Ralstonia pickettii* growth conditions: The *Ralstonia pickettii* strain (Gram-negative, Order *Burkholderiaceae*, Family *Ralstoniacea*) was obtained from the Pasteur Institute. *R. picketti* was spread on TCS (triptone-caseine-soja) agar. The strain was liquid cultured in Brain Heart Infusion (BHI) complete medium at 30 °C under agitation (150 rpm) and stored in 30% glycerol at −80 °C. When grown at confluence, bacteria were centrifuged (3000 rpm × 10 min) and the pellet was washed with PBS and lyophilized for further analysis or purification.

*E. coli* J5 LPS was extracted from strain ATCC 43745. Batch: 61792372. *Salmonella enterica* Typhimurium Rough mutant Ra strain originates from SGSC, Edmonton, AB, Canada. 

### 4.2. LPS and Lipid A Preparation

The *Bordetella*, *Salmonella*, and *Ralstonia* LPSs were extracted by the Isobutyric acid/1 M ammonium hydroxide method [114]. While extracting LPS either by this method or phenol-water methods, it is recommended to always compare LPS profiles of the bacteria to those of the extracted LPS, and systematically verify that the extracted residual biomass does not contain any more material having escaped to the extraction process which would lead to molecular species segregation. 

The *Neisseria* and *Yersinia* LPSs were extracted by the Phenol–Water method as described in [113]. Briefly, cells were suspended in an equal volume of water and 90% (*w*:*v*) phenol and stirred at 60 °C for 15 min. The solution was taken to 15 °C before centrifugation (18,000× *g*, 15 min). The aqueous phase was extensively dialyzed against tap water prior to lyophilization.

All LPSs were further purified by enzymatic treatments at a final concentration of 1 µg/mL (Sigma-Aldrich, Saint Louis, MO, USA) to remove DNA, RNA, and proteins. They were also extracted with a mixture of solvents (chloroform:methanol, *v*:*v*) to remove phospholipids and lipoproteins. The degree of purity of each sample to be used for biological studies was checked by UV spectrometry, SDS-PAGE, TLC and amino acid analyses for the detection of amino acid components as markers of peptides and proteins, and for Muramic acid as a marker for eventual peptidoglycan contaminants [115]. 

Lipid A was obtained either by the rapid TEA–citric acid method, or by our innovative and widely applied direct rapid micro hydrolysis of bacteria for MALDI-MS bacterial detection [116]. Finally, the insoluble lipid A was extracted twice in a 25–100 µL mixture of chloroform:methanol:water (3:1.5:0.25, *v*:*v*:*v*).

In all experiments presented in this paper, lipid A molecular species appearing in the spectra do not originate from MS fragmentation. This was earlier demonstrated in *E. coli* and Salmonella lipid A characterization using PDMS [117] and verified in MALDI-MS.

### 4.3. Sequential Ester-Linked Fatty Acids’ Release by Mild Alkali Treatments

This treatment was used to establish the lipid A acylation patterns [94]. For the first-step liberation of primary ester-linked fatty acids, lipid A (200 µg) was suspended at 1 mg/mL in 28% ammonium hydroxide and stirred for 5 h at 50 °C. The solutions were dried under a stream of nitrogen, the residues taken up in a mixture of chloroform: methanol: water (3:1.5:0.25, *v*:*v*:*v*) followed by MALDI-MS analysis. In order to liberate the more resistant secondary ester-linked fatty acids due to steric hindrance, the resulting lipid A was suspended in 200 µL of 41% methylamine and stirred for 5 h at 37 °C. The resulting samples were dried under a stream of nitrogen, and the residues were taken up in a mixture of chloroform, methanol, and water (3:1.5:0.25, *v*:*v*:*v*) and followed by MALDI-MS.

### 4.4. LPS Quantification by 3-Hydroxy Fatty Acids’ Dosage

*β*-hydroxylated fatty acids were released from LPSs and quantified by LC-MS^2^. An equivalent volume of fuming HCl was added to LPS solutions and heated at 90 °C for 4 h. Hydrolyzed fatty acids were recovered with hexane before vacuum evaporation. The enriched fatty acid fraction was resuspended in a mixture of 40% ammonium acetate at 5 mM pH 5.0 and 60% acetonitrile/acetate ammonium at 5 mM pH 7.3.

This solution was injected into an Infinity 1200 HPLC binary system (Agilent, Santa Clara, CA, USA) with a QQQ 6460 triple quadruple mass spectrometer (Agilent, Santa Clara, CA, USA) for MS^2^ detection [107]. 

### 4.5. Chemical Analyses

Fatty acids were analyzed as in [60]. Briefly, LPSs (1 mg) were hydrolyzed by strong acid treatment with 4 M HCl, for 2 h at 100 °C with 20:0 (20 µg) as an internal standard, extraction with ethyl acetate, and esterification with diazomethane. GC-MS analysis was performed using a Finnigan MAT 95S mass spectrometer (Finnigan MAT GmbH, Bremen, Germany). 

### 4.6. SDS–Polyacrylamide Gel Analysis of LPS

Fifteen percent acrylamide gel was used, and 0.2 μg of LPS was loaded onto the 4% stacking gel. The LPS preparation, electrophoresis process, and silver nitrate staining were performed as previously described [55].

### 4.7. MALDI Mass Spectrometry

Positive- and negative-ion MALDI mass spectra were acquired on a Shimadzu Axima Performance system (Shimadzu Kratos, Manchester, UK) operated in linear mode with delayed extraction.

The ion-accelerating voltage was set at 20 kV. Dihydroxybenzoic acid (DHB) (Sigma Chemical Co., St. Louis, MO, USA) dissolved in 0.1 M citric acid was used as a matrix [61]. A few micrograms of lipid A were dissolved in a mixture of chloroform: methanol: water (3:1.5:0.25, by vol.) at 1 µg/µL and desalted with a few grains of ion-exchange resin (Dowex 50W-X8) (H^+^) in an Eppendorf tube. A 1 µL aliquot of the solution (50 µL) was deposited on the target and covered with the same amount of the matrix suspended at 10 mg/mL in the same mixture of solvents. Different sample-to-matrix ratios were tested. *B. pertussis* and *E. coli* lipid As were used as an external standard.

### 4.8. LAL Detection Test

LAL tests for estimating samples levels of EUs were performed at Charles River (Ecully, France) employing the kinetic chromogenic method. LPS powders were reconstituted with pyrogen-free water and properly diluted to match the reading frame of the LAL assay.

### 4.9. TLR-4 Reporter Assays

Human HEK-Blue reporter cells expressing human TLR4 and coreceptors (CD14 and MD2) were used to measure TLR4 stimulation by monitoring the activation of nuclear factor-κB using secreted embryonic alkaline phosphatase (SEAP) assays (InvivoGen, Toulouse, France). SEAP assays were performed to evaluate the extent to which TLR4 signaling was activated in HEK-Blue reporter cells by the tested molecules. HEK cells were cultured according to the manufacturer’s instructions in DMEM with 10% FBS (Thermo Fisher Scientific, Waltham, MA, USA) and selection antibiotics (InvivoGen) in a humidified 5% CO_2_ incubator at 37 °C. Cells were harvested when they were 70–80% confluent and plated at a density of 1.4 × 10^5^ cells/well in flat-bottom 96-well microtiter plates. Cells were then exposed to tested molecules for 18–24 h. Cell supernatants were harvested and analyzed for NFκB activity via SEAP production following the manufacturer’s instructions using the QUANTIBlue kit (InvivoGen). SEAP activity was assessed by reading the optical density (OD) at 620 nm with a microplate reader (Thermo Fisher Scientific). Data are expressed as the fold change in OD over vehicle-treated cells. Dose curves were performed with each tested molecule using one-log or half-log step dilutions from a maximum concentration of 0.1 or 1 μg/mL to assess dose–response profiles.

The potency of candidates was determined using GraphPad Prism 10.0 (GraphPad Software, San Diego, CA, USA) to calculate the EC50 values. EC50 values were normalized on the EC50 of the standard (*E. coli* O55:B5). The EC50 ratio was calculated using this equation: EC50 ratio = EC50standard/EC50samples. This equation allows us to compare the results obtained in different runs of measurements.

## 5. Conclusions

LPS molecules are complex and highly different molecules depending on their genera and even species origins. They are heterogeneous and should not be expected resemble the classical lipid A structure first determined for *E. coli* and *Salmonella*. 

Growth conditions such as temperature and colony type (biofilm) play a critical role in the precise LPS expressed [88]. Therefore, one cannot completely rely on the structures described in the literature for bacteria grown in optimal laboratory conditions. LPS structural variability was illustrated here with examples of two *Yersinia* strains grown at different temperatures and for LPSs from other different genera.

Testing biological activities of such diverse and variable LPS structures in LAL and hTLR4 HEK-Blue assays, we demonstrated the following: (1) the two tests respond differently to the same structures; (2) LAL has the highest overall sensitivity but does not predict the toxicity of LPS molecules; (3) hTLR4 HEK-Blue is more specific for potential LPS toxicity; (4) both methods can be applied for LPS quantification but would require the introduction of specific LPS standards with appropriate structures.

Some LPS detection methods cannot be used in biological matrices, like blood or feces. In such a case, the chemical method for quantification of LPS markers like 3-OH FAs measured by LC-MS^2^ can be applied and compared to LPS structures when possible.

MALDI-MS is another powerful tool to precisely characterize LPS structures and therefore their activities when sufficient material is available. Micro-methods developed in our lab allow for the profiling of lipid A molecular species from bacterial samples as small as 1 × 10^4^ CFU as well as directly from certain biological samples [69]. 

Based on the data presented, research involving LPS molecules should consider high variability and diversity; and LPS detection, especially when dependent upon biological assays, would benefit from the use of at least two different methods.

## Data Availability

Data are contained within the article.

## Abbreviations

DHB	2,5-dihydroxybenzoic acid
EU	Endotoxin unit
FA	Fatty Acid
GlcN	Glucosamine
HEK	Human embryonic kidney
IL	Interleukin
LC-MS^2^	Liquid Chromatography–Mass Spectrometry
LAL	Limulus amoebocyte Lysate
LOD	Limit of detection
LOS	Lipooligosaccharide
LPS	Lipopolysaccharide
MALDI	Matrix-assisted laser desorption ionization
MAT	Monocyte Activation Test
MD-2	Myeloid Differentiation Protein-2
OMV	Outer membrane vesicle
PBS	Phosphate Buffer Saline
PEA	Phosphoethanolamine
PS	Polysaccharide
rFC	Recombinant factor C
RPT	Rabbit pyrogen test
RSE	Reference Standard Endotoxin
TLR	Toll Like Receptor
